# Feasibility of Cancer Detection Through X-Ray Diffraction of Human Nails

**DOI:** 10.3390/cancers18203270

**Published:** 2026-10-10

**Authors:** Andre L. C. Conceiçao, Alexander Alekseev, Viacheslav Shcherbakov, Dmitriy Shestakov, Uldana Sagyngali, Kairat Shakeev, Assylbek Zeinidenov, Assel Zhumina, Aitbek Aimukhanov, Galym Turgambekov, Anna Kryzhanovskaya, Keith Rogers, Lev Mourokh, Pavel Lazarev

**Affiliations:** 1 Deutsches Elektronen-Synchrotron DESY, 22607 Hamburg, Germany; 2 Bragg Analytics, Inc., 1900 S. Norfolk Street, #350, San Mateo, CA 94403, USAk.d.rogers@cranfield.ac.uk (K.R.); plazarev@matur.co.uk (P.L.); 3 Matur UK Ltd., 5 New Street Square, London EC4A 3TW, UK; aalexeev@matur.co.uk (A.A.); viacheslav.s@matur.co.uk (V.S.); 4 Scientific Center of Nanotechnology and Functional Nanomaterials, Buketov Karaganda National Research University, 28 University Street, Karaganda 100028, Kazakhstan; assylbek.zeinidenov@knus.edu.kz (A.Z.); zhumina.a@karnu-buketov.edu.kz (A.Z.);; 5 Surgery Department, Regional Oncology Center of Karaganda, 189 Amanzholov Street, Karaganda 100019, Kazakhstanshakeev@qmu.kz (K.S.); 6 Department of Surgical Diseases, Karaganda Medical University, 40 Gogol Street, Karaganda 100012, Kazakhstan; 7 Department of Science and Commercialization, Kazakh National University of Sports, 15 Karkaraly Street, Astana 010000, Kazakhstan; 8 Biology-Geography Faculty, Buketov Karaganda National Research University, 28 University Street, Karaganda 100028, Kazakhstan; 9 Department of Ecology and Assessment, Karaganda University of Kazpotrebsoyuz, 9 Academic Street, Karaganda 100009, Kazakhstan; 10 Faculty of Engineering and Applied Sciences, Shrivenham Campus, Cranfield University, Swindon SN6 8LA, UK; 11 Physics Department, Queens College, City University of New York, 65-30 Kissena Blvd, Flushing, NY 11367, USA

**Keywords:** X-ray diffraction, cancer detection, structural biomarkers, keratin, machine learning, Fourier coefficients

## Abstract

**Background/Objectives**: Establishing biomarkers is crucial for early cancer detection. Structural biomarkers can be revealed by noninvasive X-ray diffraction. We applied this approach to human nails and observed structural changes in the major nail tissue component, keratin, which coincided with a cancer diagnosis. **Methods**: X-ray diffraction patterns were obtained from 230 nail samples (control/cancer split: 92/138) belonging to 133 patients (48/85) in wide (WAXS) and small (SAXS) ranges of scattering angles. For both ranges, we obtained one-dimensional scattering profiles in meridional and equatorial directions. We examined these profiles using machine-learning techniques such as logistic regression and linear discriminant analysis. **Results**: We obtained good classification metrics that allow us to distinguish participants with cancer from controls. The results are similar for both machine-learning methods. Combining all four profiles (WAXS meridional, WAXS equatorial, SAXS meridional, and SAXS equatorial) produced a sensitivity of 0.753 and a specificity of 0.771 for the Linear Discriminant Analysis at the Youden point. For the limited set of patients of the same age, we showed that the patterns can still be distinguished, supporting our hypothesis that the observed aberrations are cancer-induced. **Conclusions**: Our exploratory research demonstrated the feasibility of cancer detection using the X-ray diffraction of human nails.

## Simple Summary

Cancer screening using noninvasively obtained biomarkers can revolutionize diagnostics. In this sense, nails are particularly attractive because they can be collected during routine hygiene procedures and transported to a laboratory in a simple package without damage. Following our previous work in a canine model, we performed pilot studies of X-ray scattering from human nails. We analyzed the resulting patterns using a machine-learning technique and achieved high accuracy in distinguishing samples from healthy individuals and samples from cancer patients. This approach can lead to early cancer detection and a decrease in cancer-caused mortality rates.

## 1. Introduction

Cancer remains the most challenging problem facing the global public health system. In 2026, approximately 2,114,850 new cancer cases and 626,140 cancer deaths are projected to occur in the United States [1]. Mortality rates are trending downward, at least in upper-middle- and high-income countries [2]. Such a tendency is caused by very early cancer detection, before the onset of symptoms, and the established screening systems for several types of cancer (breast, stomach, or colon). However, such screening procedures are not possible for other cancer types, and, moreover, they are not accessible in low-income communities.

Establishing a biomarker related to a tissue that is easy to collect, store, and transfer to a laboratory facility is paramount. In that sense, nails and hair are specifically appealing. Nail clipping and hair cutting are routine hygienic procedures, so specimens can be sampled noninvasively by untrained personnel and are relatively stable. The number and diversity of diseases in which nails have been the subject of focused research for diagnostic and prognostic biomarkers is remarkable [3]. It includes, in particular, diabetes [4], chronic kidney disease [5], gout [6], and Alzheimer’s [7,8]. A similar list of uses for hair as a biomarker is no less impressive [9]. In relation to cancer, nails can contain traces of cancer-induced aberrations in lipid metabolism [10,11] or cancer-associated low selenium levels [12,13,14].

In the present paper, we address another type of biomarker related to the molecular structure of the main component of nails, keratin. Structural biomarkers can be revealed by X-ray scattering at small (SAXS) and wide (WAXS) angles. Previously, cancer-induced modifications to collagen fibril repeat distances [15,16,17,18], alterations to the amorphous scattering profile [17,18,19,20], and disruption to triglyceride molecular packing [21,22] were observed in the SAXS region. WAXS addresses modifications to lipid and aqueous components [23,24,25,26,27,28].

X-ray diffraction has been used to characterize the molecular architecture of keratinized tissues for over seven decades. Hard α-keratin, the structural protein of hair and nails, is built from α-helical intermediate filaments embedded in a sulfur-rich matrix, giving rise to a characteristic fiber-diffraction signature: a meridional reflection near 0.515 nm (momentum transfer *q* = 12.2 nm^−1^) and a strong equatorial reflection near 0.98 nm (momentum transfer *q* = 6.4 nm^−1^) arising from the coiled-coil and its lateral packing, together with a set of low-angle equatorial maxima that encode the diameter and packing of the microfibrils at *q* = 0.73 nm^−1^, as well as the meridional diffraction profile of the keratin intermediate filament (7th reflection order (*q* = 0.98 nm^−1^)) [29,30,31,32]. These features are stable and well understood, which is precisely what makes keratinized tissues attractive as a diagnostic substrate, because hair and nail structure reflects the interplay between the follicle or matrix and its systemic environment [33]. Nails are particularly appealing in this respect, as clippings are collected non-invasively, are chemically robust, and can serve as stable bioarchives of protein and elemental composition [34].

The SAXS region around *q* = 1.32 nm^−1^ is of special interest, as it contains several features of different origin. The existence of a diffraction ring at this value of the momentum transfer was attributed to “lipid crystals” [35]. Later, it was shown that one of the peaks produced by lateral intermediate-filament packing is located there [36,37]. This diffraction ring has also been attributed to proteoglycan structures [38]. The relationship between this feature and structural changes associated with cancer status in the keratin molecules of nails and hair has been the subject of heated debate. In a series of publications [39,40,41,42,43], it was argued that the appearance of this ring manifests cancer development. However, these measurements and their interpretations were disputed [44,45,46]. It should be emphasized that we did not observe any specific cancer-induced features in this region, and our SAXS and WAXS results complement each other.

The cancer-induced modifications to keratin diffraction patterns in the WAXS region were examined in [47,48] in the canine model. Excellent metrics were obtained using the binary cancer/non-cancer classification for 945 nail samples from 266 dogs, with 104 of them diagnosed with cancer.

In this work, we conducted pilot studies on human nail samples from participants with cancer and controls at the synchrotron facilities in DESY, Hamburg, Germany. We analyzed the diffraction patterns using machine-learning methods and achieved strong classification metrics, indicating the feasibility of cancer screening using human nails.

## 2. Materials and Methods

### 2.1. Collection of Samples

Nail plate samples were obtained from the Multidisciplinary Hospital No. 3 in Karaganda, Kazakhstan. The study sample included adult participants from various clinical groups, including patients with malignant neoplasms at different sites, individuals with benign conditions, and healthy volunteers who served as the control group. The demographic, clinical, and diagnostic information for this cohort is included in the Supplementary Materials. Biological material was collected according to a standardized protocol: using sterile instruments, a fragment of the free edge of the nail plate, approximately 1–2 mm in size and up to 1 cm in length, was clipped. The standard procedure was to take two samples from each patient from two adjacent index fingers. A few patients had 3 samples, with an additional sample from another finger. The table with the sample statistics is included in the Supplementary Materials. After collection, the samples were labeled, placed in individual bags, then in separate containers, and finally in a common transport container, providing triple protection during air transportation. Before transportation and subsequent preparation for analysis, the samples were stored at room temperature.

Material collection was performed only after obtaining informed consent from the study participants. Before inclusion in the study, each participant received oral and written information about the aim of the study, the nature of the procedure, the intended further use of the collected nail samples for scientific research, and measures to ensure confidentiality of personal data. Participants were informed that participation in the study was voluntary and that they could withdraw at any stage without giving a reason and without any consequences.

### 2.2. Preparation of Samples

**Figure 1:**
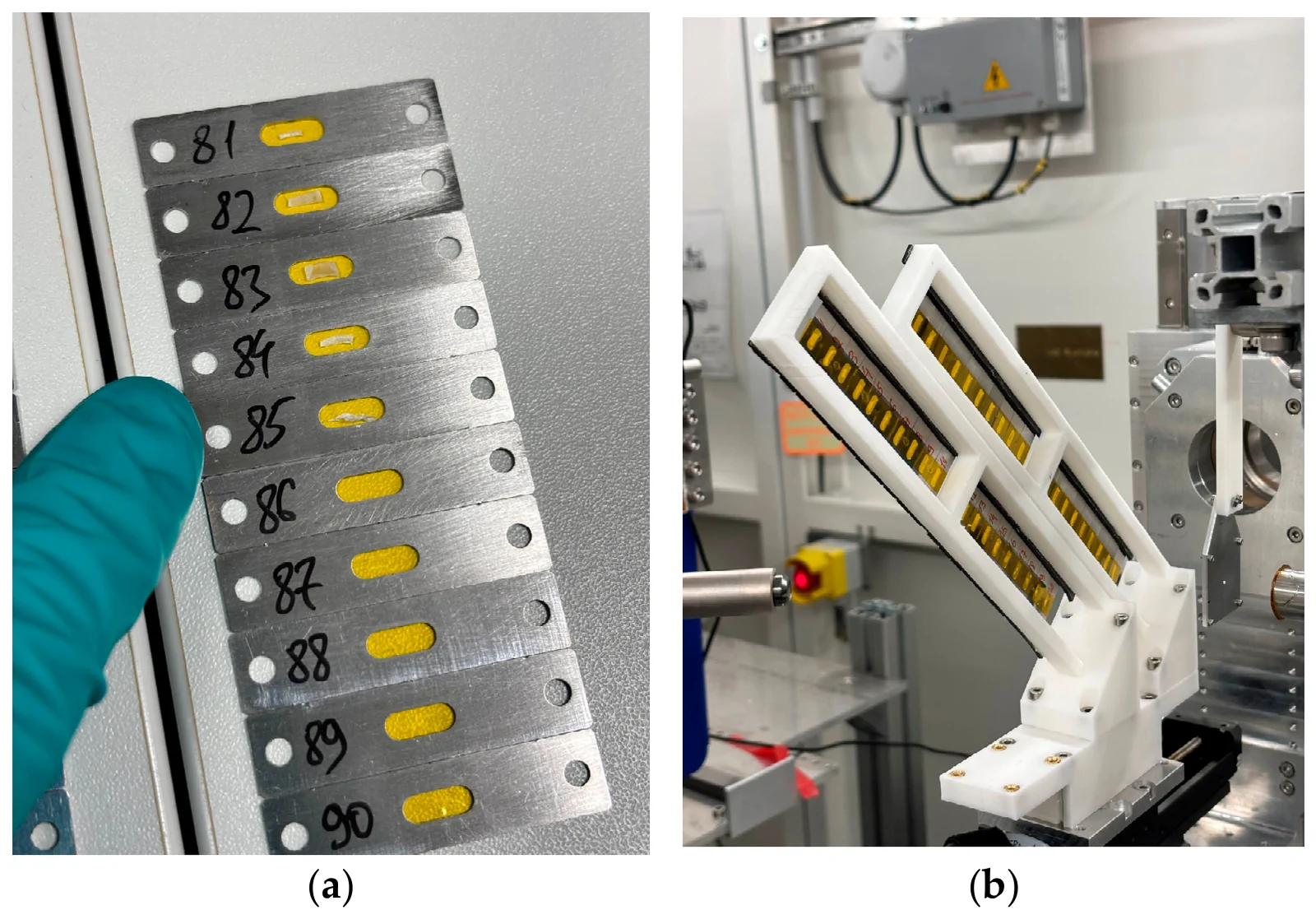
Photos of holders for nail samples: (**a**) set of 10 metal plates with a window of approximately 2 × 4 mm, bonded with Kapton tape. Note: samples were covered with Kapton film on both sides for XRD measurements; (**b**) plastic towers holding two sets of metal plates each, installed at a 45-degree angle.

Nail clipping samples were cut into approximately 1 × 2 mm fragments. Each nail fragment was placed horizontally between two layers of Kapton tape in a metal sample holder, as shown in Figure 1a. For the measurement, the packs of metal sample holders were mounted on the tower and positioned in front of the incident X-ray beam at a 45-degree angle (Figure 1b). This orientation ensured that both the meridional and the equatorial α-keratin reflections fell within the accessible azimuthal window of the WAXS detector, which was approximately 120° in the current experiment.

### 2.3. Data Acquisition

Combined small- and wide-angle X-ray scattering (SAXS/WAXS) measurements were performed at the SAXSMAT beamline P62 of the PETRA III storage ring (DESY, Hamburg, Germany) [49]. A parallel monochromatic X-ray beam with a photon energy of 11.0 keV (wavelength λ = 0.1127 nm) was selected using a Si (111) double-crystal monochromator, and higher harmonics were suppressed by a B_4_C double-mirror system. The beam was defined by slits to 200 × 200 µm^2^ at the sample position.

**Figure 2:**
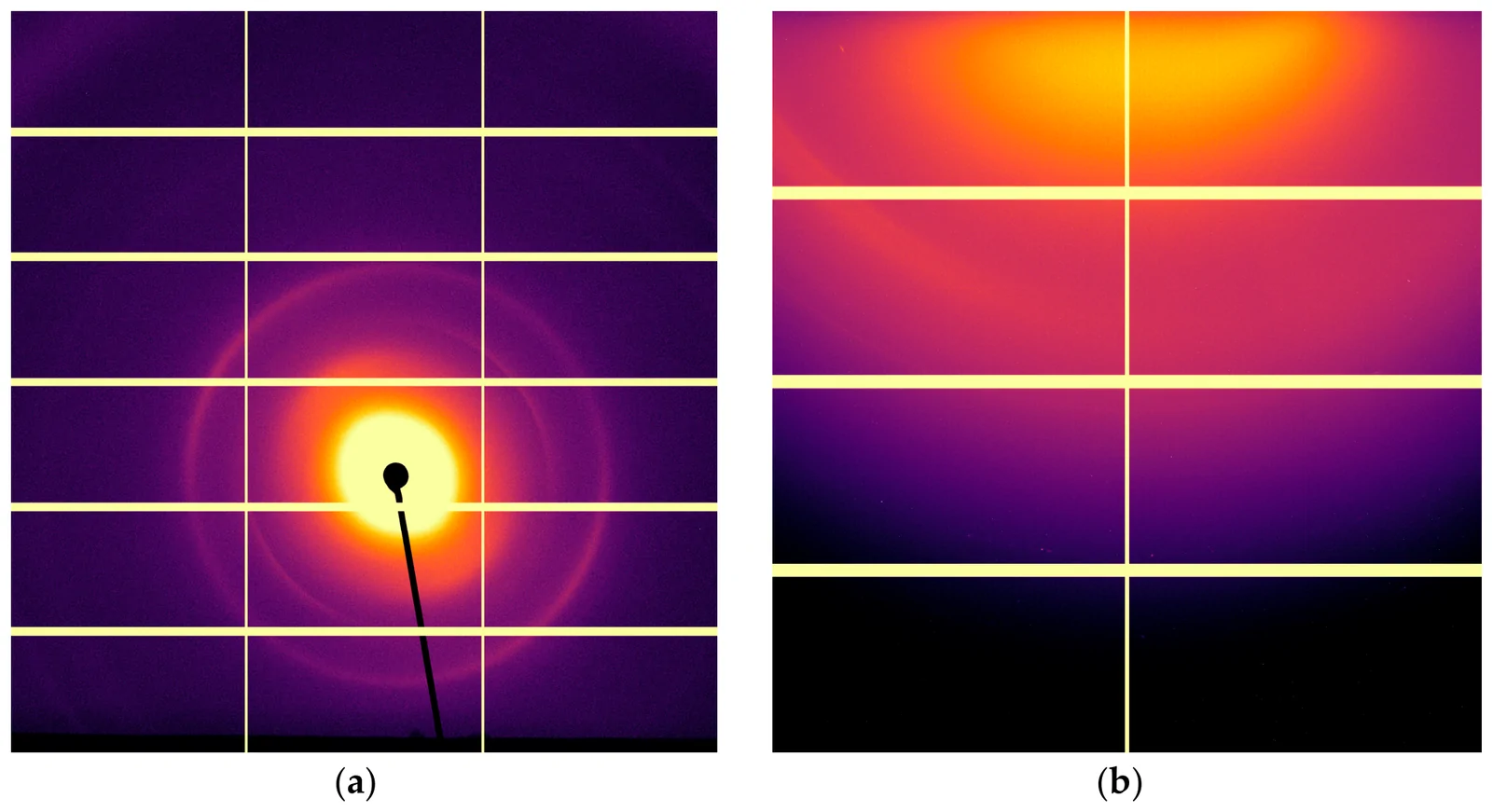
Diffraction patterns observed in (**a**) SAXS and (**b**) WAXS detectors.

SAXS and WAXS patterns were recorded simultaneously using two-dimensional single-photon-counting detectors, a DECTRIS Eiger2 X (Baden-Daettwil, Switzerland) 9M for SAXS and a DECTRIS Eiger2 X 4M for WAXS. The SAXS detector was placed at a sample-to-detector distance of 2.85 m and was operated under vacuum, whereas the WAXS detector and the sample were kept in air, with the WAXS detector positioned at 0.40 m from the sample. This configuration provided a momentum-transfer range of 0.09 nm^−1^ < *q* < 3.85 nm^−1^ for SAXS and 4.8 nm^−1^ < *q* < 26.4 nm^−1^ for WAXS, where *q* = 4π sin(*θ*)/*λ*, 2*θ* is the scattering angle, and *λ* is the X-ray wavelength. The sample-to-detector distances and *q*-scales were calibrated using silver behenate (AgBh) for the SAXS detector and α-Al_2_O_3_ for the WAXS detector. Raw SAXS and WAXS images are shown in Figure 2.

The intensities of the primary and transmitted beams were monitored by an ionization chamber placed upstream of the sample and by an active beamstop (6.0 mm diameter) mounted in front of the SAXS detector, respectively. For each sample, 20 data points were acquired on a 4 × 5 grid, with 300 µm spacing between adjacent points and an acquisition time of 1 s per point. The acquired SAXS and WAXS data were subsequently corrected for incident-beam intensity fluctuations and sample self-absorption using the ionization chamber and beamstop readings, respectively. Because the attenuation of a given point depends on the amount of material traversed by the beam, this point-by-point correction also accounts for the variability in thickness across the nail clipping and between samples. We performed background measurements using an identical sample holder, prepared with the same two layers of Kapton tape but without a nail fragment, and mounted on the same tower at the same 45° orientation as the samples. This ensured the background measurement reproduced the full scattering contribution of the air path, the Kapton windows, and the metal holder in the same geometry used for the samples. For the nail clippings, we measured the empty holder at 20 scan points, but with an exposure time three times longer per point to reduce statistical noise in the background estimate. We azimuthally regrouped and averaged the 20 background patterns the same way as the sample data, yielding one averaged background profile for each azimuthal sector. We rescaled these averaged background profiles to the acquisition time used for the samples and subtracted them from the corresponding sample SAXS and WAXS profiles.

### 2.4. Data Analysis

#### 2.4.1. Image Pre-Processing

The acquired two-dimensional scattering patterns were reduced through an in-house Python (3.11.7) processing pipeline comprising several steps. Immediately after acquisition, each SAXS and WAXS pattern was azimuthally regrouped into a two-dimensional intensity map as a function of azimuthal angle and momentum transfer, *I*(*χ*, *q*), using a script based on the pyFAI package (2024.5.0). The SAXS patterns were binned into 16 azimuthal sectors over 800 *q*-bins, and the WAXS patterns into 6 azimuthal sectors over 1500 *q*-bins.

Because keratin scattering patterns are anisotropic, we performed an orientation analysis of the azimuthal intensity distribution to separate meridional and equatorial contributions. For SAXS, the fiber orientation was determined from the azimuthal intensity at *q* ≈ 0.94 nm^−1^, corresponding to the 7th-order meridional reflection of the ~47 nm axial repeat of the keratin intermediate filaments, with the azimuth of maximum intensity defining the meridional direction. For WAXS, we determined the orientation analogously at *q* ≈ 12.1 nm^−1^. Based on the retrieved orientation, we split both the SAXS and WAXS maps into meridional and equatorial one-dimensional profiles, for a total of four profiles for each measurement.

For each sample, we averaged the meridional and equatorial profiles from the 20 (4 × 5) scan points to obtain one representative meridional and one representative equatorial profile (for both WAXS and SAXS). Before averaging, we rejected outlier profiles using a robust criterion: any profile whose deviation from the group median exceeded 3.5 times the robust median deviation was discarded. The resulting participant flow diagram is included in the Supplementary Materials.

#### 2.4.2. Machine-Learning Classification Procedure

The dataset contains 230 samples (control/cancer split: 92/138) from 133 participants (48/85), with four profiles for each sample. We explored two approaches for the analysis of the curves and cancer/control classification: (i) Fourier representation and (ii) area under the curve. For (i), we represented the curves in terms of the Fourier expansion and exploited the first 10 complex coefficients, yielding 19 parameters per profile: 10 magnitudes and 9 phases. We used the NumPy library to obtain the Fourier coefficients. For (ii), the WAXS region from 5.095 to 24.327 nm^−1^ was split into four equal fragments. The SAXS region was split into four fragments: 0.092–0.671, 0.671–1.25, 1.25–1.4, and 1.400–1.719 nm^−1^. The third region is of special interest because it includes a possible feature at *q* = 1.32 nm^−1^, previously implicated in cancer detection [39,40,41,42,43,44,45,46].

We implemented a supervised classification algorithm to evaluate the diagnostic performance of the data. Given the small dataset size, we did not use a single test split. Instead, to assess the discriminative power of the described approach, we performed repeated stratified cross-validation. We estimated classification performance using repeated stratified grouped k-fold cross-validation: 5 folds repeated 20 times, producing 100 train/test splits. The critical design constraint is that all measurements from the same patient were assigned entirely to either the training or the test set—never split between them [50]. This patient-level grouping prevents information leakage between correlated measurements from the same individual and ensures that reported metrics reflect performance on genuinely unseen patients. Class balance was preserved at the patient level across folds. Each sample received a probability score each time it appeared in the test fold—once per repeat, for a total of 20 scores per sample. We averaged these 20 scores to produce one stable out-of-fold probability estimate per sample. This averaging reduces variance from any particular fold assignment and produces a single unbiased probability for each sample that can be used for downstream analysis without refitting the model.

In this work, we used Logistic Regression (LR) with elastic net regularization [51] and Linear Discriminant Analysis (LDA) with Ledoit-Wolf regularization [52] from the Scikit-learn library to obtain performance metrics. The primary metric was the area under the receiver operating characteristic curve (AUC ROC), with the confidence interval (CI) computed by the DeLong analytical method [53]. We also obtained specificity and sensitivity at the Youden operating point [54], with CI calculated by the Wilson score [55].

Hyperparameter optimization (HPO) runs inside each outer fold via a nested 3-fold cross-validation on the training split. A randomized search (RandomizedSearchCV, 50 iterations) samples parameter combinations from predefined distributions and selects the combination that maximizes mean AUC across the 3 inner folds. The selected parameters are then used to train the final model on the full outer training set, which is evaluated on the held-out outer test fold. Two HPO parameters are tuned for LR: inverse regularization strength C (sampled from loguniform(1 × 10^−3^, 1 × 10^2^)) and l1_ratio, which is a mixing parameter between L1 and L2 regularizations (sampled uniformly in [0, 1]). No HPO is needed for LDA, since the regularization adapts to each training fold automatically.

We report results at the sample and patient levels. Sample scores were aggregated per patient using four methods: arithmetic mean, median, logit-mean, and maximum. Details of the aggregation procedure are provided in [56]. Within each cross-validation fold, we selected the aggregation method using only the training patients and then applied it to the held-out test patients; we chose the method that yielded the highest patient-level AUC on the training set. We computed final patient-level metrics from the out-of-fold test predictions accumulated across all folds and repeats. For LDA, raw decision function scores were mapped to probabilities via the sigmoid function before aggregation.

## 3. Results

### 3.1. Scattering Profiles

**Figure 3:**
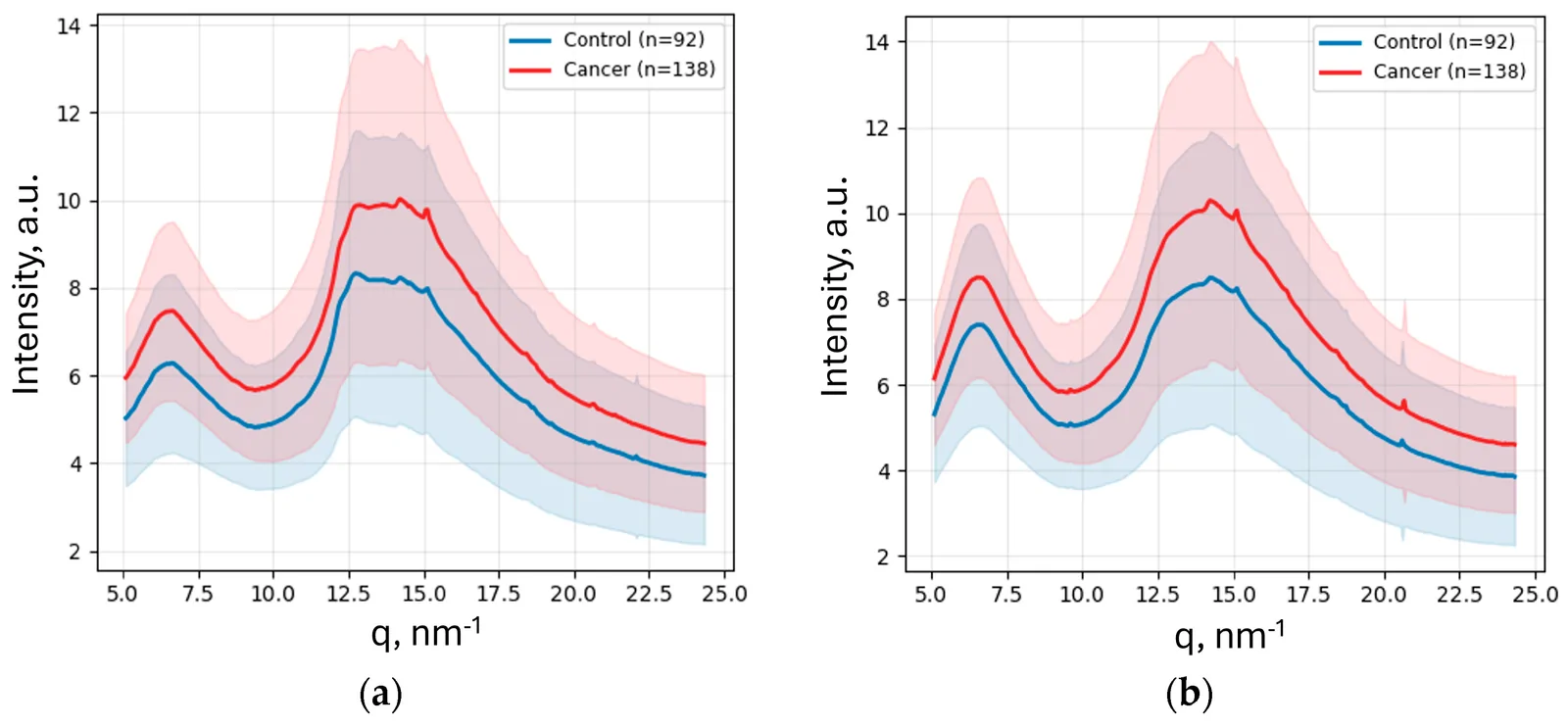
Scattering profiles in the WAXS region in (**a**) meridional and (**b**) equatorial directions.

**Figure 4:**
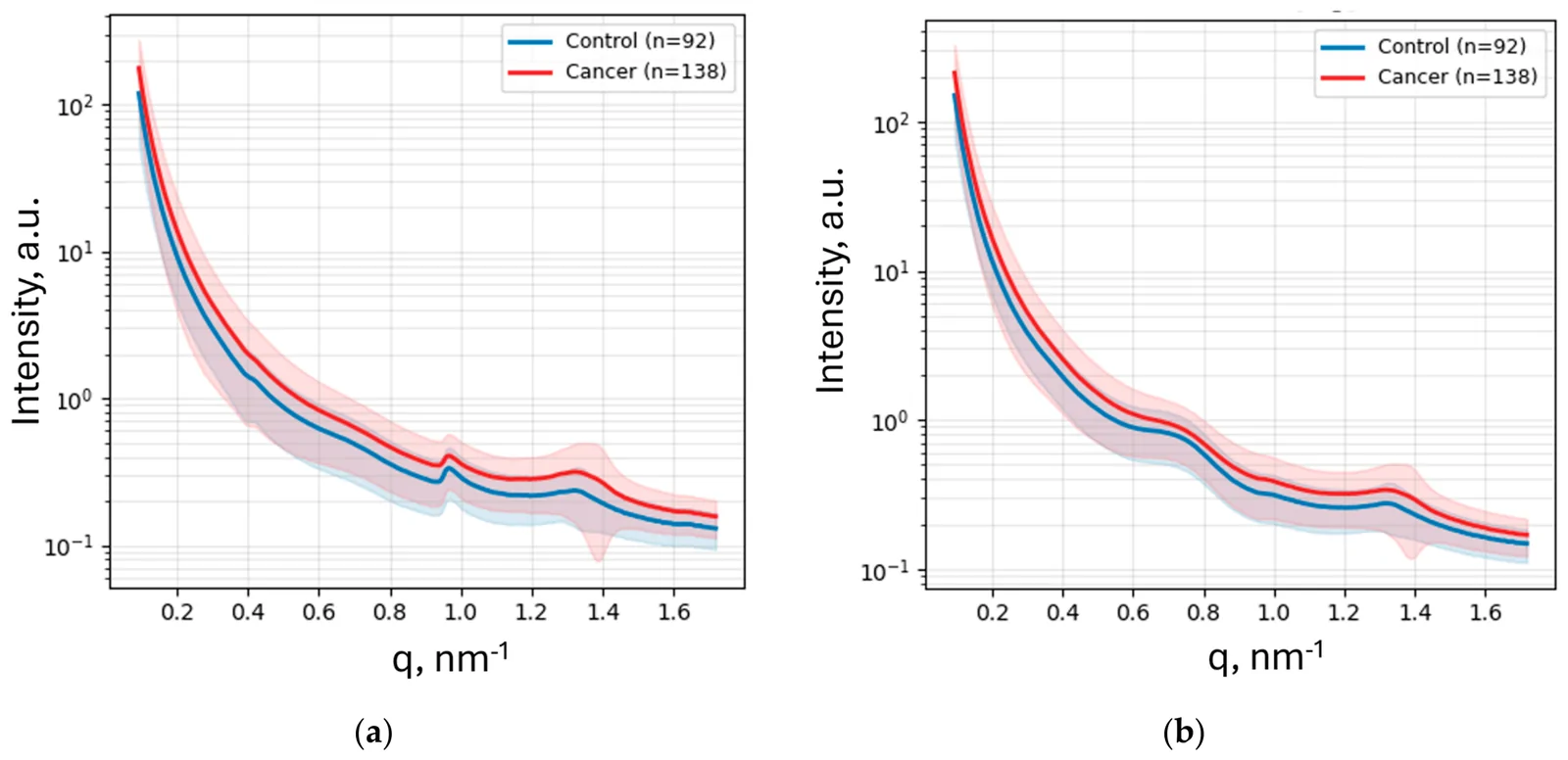
Scattering profiles in the SAXS region in (**a**) meridional and (**b**) equatorial directions.

The scattering profiles, i.e., the dependence of intensity on momentum transfer in the meridional and equatorial directions, averaged over cancerous and control samples, are shown in Figure 3 for WAXS and in Figure 4 for SAXS. The full sets of individual profiles are available in the Supplementary Materials.

In WAXS (Figure 3), both meridional and equatorial profiles exhibit a series of overlapping lipid maxima above 14 nm^−1^. In the meridional profile, there is an additional feature at 12.2 nm^−1^, associated with the keratin coiled-coil. The peak at 6.5 nm^−1^, related to the inter-helix distance in the keratin dimer, was expected to appear only in the equatorial profile but appeared in both because this feature became extended and ring-like in most of our samples. In SAXS, as expected, the meridional profile contains the peak around 0.98 nm^−1^, related to the 7th reflection order of the keratin intermediate filament, and the equatorial profile exhibits a maximum near 0.73 nm^−1^, associated with the diameter and packing of the microfibrils. A maximum near 1.32 nm^−1^ appeared in both profiles.

### 3.2. Fourier Coefficients Analysis

**Figure 5:**
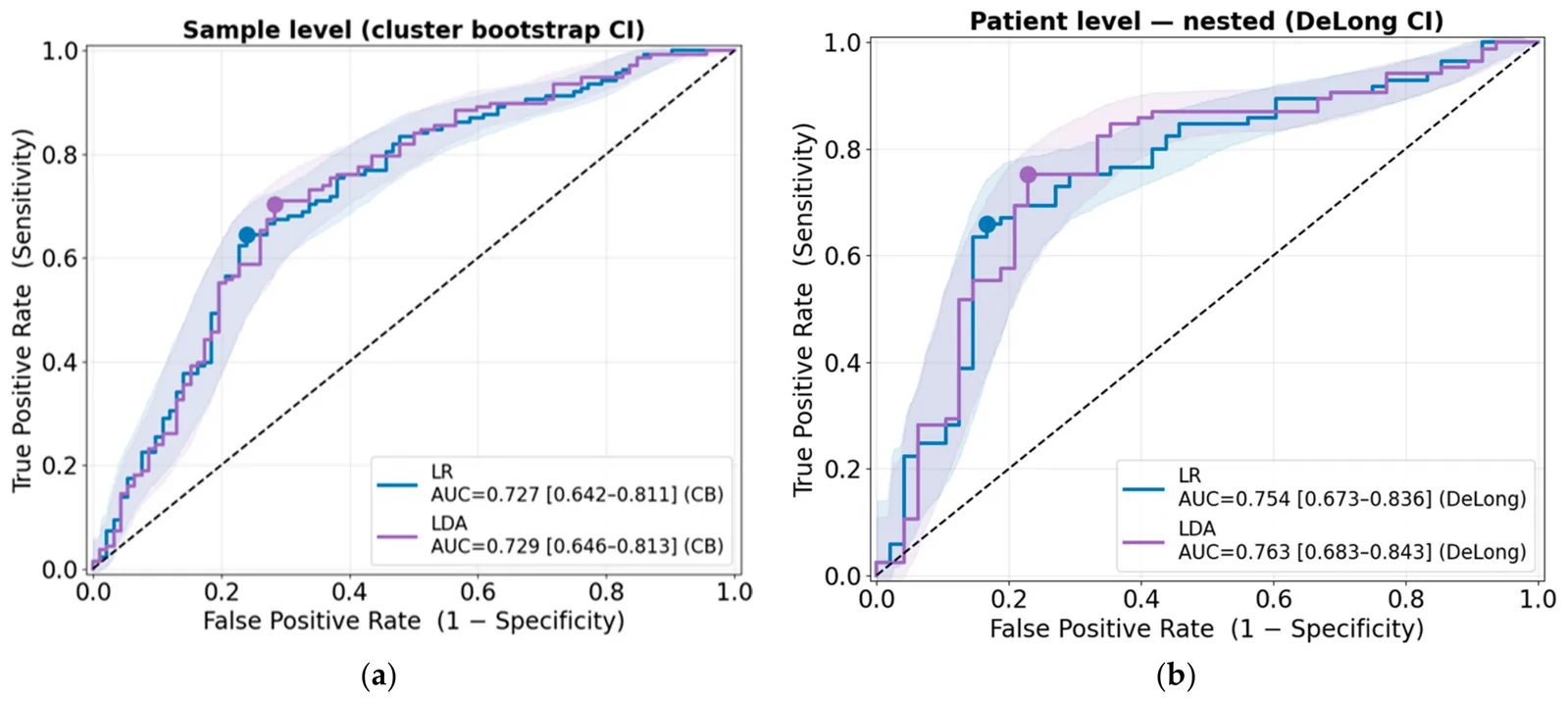
Receiver operating characteristic curves at (**a**) sample and (**b**) patient levels with 95% confidence intervals. The blue and purple curves are related to LR and LDA, respectively. The dots indicate the Youden points. Confidence intervals are obtained by CB for samples and by the DeLong method for patients.

The obtained scattering profiles (WAXS-meridional, WAXS-equatorial, SAXS-meridional, and SAXS-equatorial) were evaluated using the Fourier expansion, and the obtained sets of complex Fourier coefficients were used for the binary cancer/non-cancer classification. Figure 5 shows the ROC curves based on all four sets at the sample and patient levels using both LR and LDA.

LR classification for samples produced an AUC-ROC of 0.727 [0.642; 0.811], where the numbers in the square brackets indicate the 95% confidence interval, which improved to 0.754 [0.673; 0.836] after the sample-to-patient aggregation. LDA provided similar metrics of 0.729 [0.646; 0.813] and 0.763 [0.683; 0.843], respectively. The classification improvement after aggregation can be seen in Table 1, which includes the numbers of true positives (TP), true negatives (TN), false positives (FP), and false negatives (FN).

The number of false negatives is quite large. False negatives can provide inappropriate reassurance and delay further investigation or treatment, with consequences for prognosis. Therefore, additional studies are needed to reduce this number.

To determine the most important features for the classification, we present the metrics (AUC ROC and sensitivity/specificity at the Youden point) for individual scattering profiles and their combinations in Table 2.

This table shows that combining all profiles generated the best metrics. Adding blocks improved performance, confirming that the Fourier harmonic structure of each measurement geometry carried partially independent classification information. The four XRD geometries captured different aspects of nail tissue structure—axial periodicity, lateral packing, and long-range order—and their Fourier representations were not redundant. The meridional direction was shown to be more informative for diagnostics than the equatorial direction.

### 3.3. q-Range Analysis

An alternative approach was employed: classification based on the area under the scattering profile curves (total amount of scatter) at certain *q*-ranges. We separated WAXS and SAXS profiles (both meridional and equatorial) into four segments each, yielding 16 parameters per sample. As with the Fourier coefficients approach, we applied LR and LDA to obtain the classification metrics shown in Table 3.

The performance metrics for this approach were slightly inferior to those for the Fourier coefficients, but all main properties remained intact. Profile aggregation improved the metrics; WAXS complemented SAXS; and the meridional direction was more discriminative than the equatorial one.

We paid special attention to the *q*-range of 1.25–1.4 nm^−1^ because it includes a possible feature at *q* = 1.32 nm^−1^, previously proposed to be indicative of cancer [39,40,41,42,43,44,45,46]. Table 4 shows the classification metrics obtained using this range alone.

Performance at these momentum-transfer values alone was significantly poorer than across the full range; therefore, our results do not support the suggestions of [39,40,41,42,43], and our classification is not based on the feature at *q* = 1.32 nm^−1^.

## 4. Discussion

We collected the X-ray diffraction patterns on 230 nail samples from 133 patients, with a 48/85 healthy/cancer split. The measurements were performed in two distinct scattering-angle ranges: wide (WAXS) and small (SAXS). We chose two orthogonal directions, meridional and equatorial; therefore, we obtained four scattering profiles per measurement. Each sample was measured at 20 positions. We aggregated the results at the measurement-to-sample and sample-to-patient levels.

We used two approaches for the classification. In the first, we expanded each scattering profile in Fourier harmonics and used the complex Fourier coefficients as classification parameters. In the second, we divided each scattering profile into four segments and performed the classification based on the areas under the curve in these segments. In both approaches, we exploited two methods: logistic regression (LR) and linear discriminant analysis (LDA).

In all cases, we achieved good classification, with the best results obtained for LDA based on Fourier coefficients using all four scattering profiles. These patient-level metrics are (with 95% confidence intervals in square brackets): AUC ROC 0.779 [0.683–0.843], sensitivity 0.753 [0.652–0.832], and specificity 0.771 [0.635–0.867]. Overall, LR and LDA produced similar metrics. We used these two simple linear classifiers as a proof of principle to show that two distinct clusters exist for participants with cancer and controls. We will use more complex nonlinear classifiers for data analysis in future studies.

The WAXS and SAXS regions complemented each other, providing information over a wide range of spatial scales. At the same time, the scattering profiles in the meridional direction are better distinguished for cancer/non-cancer patients, indicating that cancer more strongly alters structures along the keratin molecules than the separation of molecules in the dimers.

**Figure 6:**
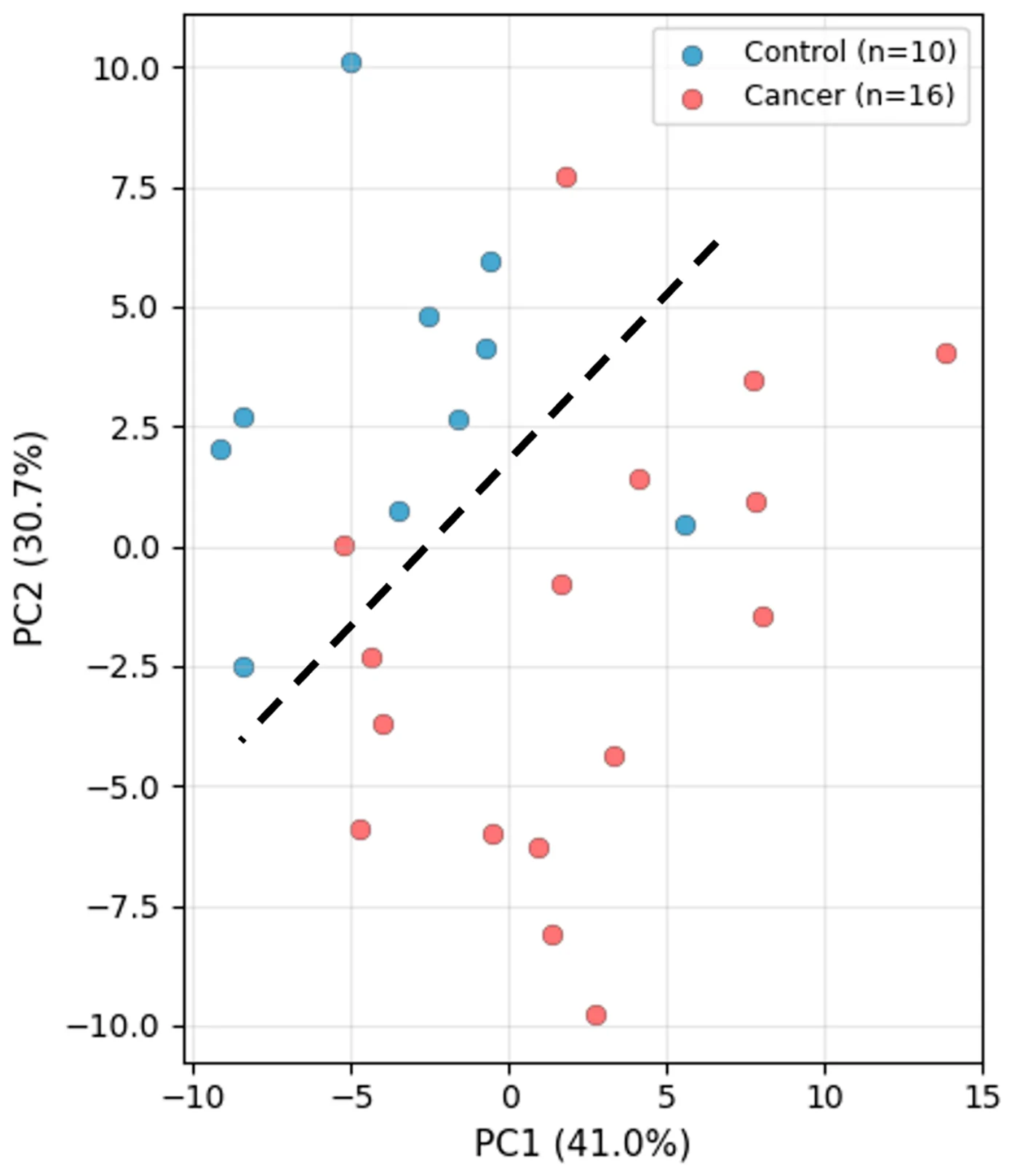
PCA-transformed data for participants with cancer and controls born in 1965–1975 within the 2D PC space.

Naturally, it remains unclear whether the observed changes in keratin are induced specifically by cancer, rather than by other pathologies or even the patients’ age. We believe comprehensive studies are necessary to fully distinguish alterations caused by different factors. However, to extract this information from our limited dataset, we performed principal component analysis (PCA) on patients born in 1965–1975. This interval has the greatest overlap, with 10 healthy and 16 cancerous samples. These numbers are not enough for a rigorous statistical analysis, but the visualization of the first two PCA components exhibits clear cluster separation, as shown in Figure 6.

If proven valid, cancer detection using the nails can revolutionize cancer screening worldwide. Nail clipping is part of standard hygiene, and samples can be routinely sent to a laboratory for analysis. As a result, health status can be continuously monitored, and if aberrations in the XRD patterns are detected, the patient can be asked to visit a medical office. Thorough studies in various locations are certainly necessary to advance this approach, but this work represents a first step in this direction.

## 5. Conclusions

In conclusion, we measured the X-ray diffraction of human nails and found that structural changes in keratin allow us to distinguish healthy samples from cancerous ones. Machine-learning analysis of the obtained scattering profiles produced strong classification metrics. While these studies are exploratory and subsequent comprehensive studies are necessary, our results represent the first step toward noninvasive cancer detection using nails, which are easy to collect, transport to the laboratory, and measure.

## Figures and Tables

**Table 1:** Statistics on classification results.

Model		TP	TN	FP	FN
LR	samples	88	70	22	50
	patients	56	40	8	29
LDA	samples	96	66	26	42
	patients	64	37	11	21

**Table 2:** Classification metrics at the patient level for specific scattering profiles and their combinations using the Fourier coefficients.

Model	Metrics	WAXS Merid	WAXS Equat	SAXS Merid	SAXS Equat	WAXS	SAXS	WAXS + SAXS
LR	AUC ROC	0.745 [0.662–0.827]	0.661 [0.568–0.754]	0.738 [0.654–0.821]	0.706 [0.618–0.794]	0.738 [0.655–0.822]	0.724 [0.639–0.81]	0.754 [0.673–0.836]
	Sensitivity	0.741 [0.639–0.822]	0.706 [0.602–0.792]	0.682 [0.577–0.772]	0.753 [0.652–0.832]	0.576 [0.470– 0.676]	0.824 [0.729–0.890]	0.659 [0.553–0.751]
	Specificity	0.729 [0.590–0.834]	0.667 [0.525–0.783]	0.750 [0.612–0.851]	0.646 [0.504–0.766]	0.854 [0.728–0.928]	0.583 [0.443–0.712]	0.833 [0.704–0.913]
LDA	AUC ROC	0.750 [0.668–0.832]	0.647 [0.553–0.742]	0.735 [0.651–0.819]	0.704 [0.615–0.792]	0.755 [0.674–0.836]	0.724 [0.639–0.810]	0.763 [0.683–0.843]
	Sensitivity	0.753 [0.652–0.832]	0.624 [0.517–0.719]	0.635 [0.529–0.730]	0.718 [0.614–0.802]	0.600 [0.494–0.698]	0.647 [0.541–0.740]	0.753 [0.652–0.832]
	Specificity	0.708 [0.568–0.818]	0.625 [0.484–0.748]	0.771 [0.635–0.867]	0.688 [0.547–0.801]	0.854 [0.728–0.928]	0.771 [0.635–0.867]	0.771 [0.635–0.867]

**Table 3:** Classification metrics at the patient level for specific scattering profiles and their combinations using the areas under the scattering profile curves.

Model	Metrics	WAXS Merid	WAXS Equat	SAXS Merid	SAXS Equat	WAXS	SAXS	WAXS + SAXS
LR	AUC ROC	0.733 [0.649–0.818]	0.623 [0.526–0.719]	0.730 [0.645–0.814]	0.697 [0.608–0.79]	0.718 [0.632–0.805]	0.738 [0.654–0.82]	0.739 [0.656–0.823]
	Sensitivity	0.776 [0.677–0.852]	0.576 [0.470–0.676]	0.671 [0.565–0.761]	0.482 [0.379–0.59]	0.765 [0.664–0.842]	0.718 [0.614–0.8]	0.647 [0.541–0.740]
	Specificity	0.625 [0.484–0.748]	0.625 [0.484–0.748]	0.771 [0.635–0.867]	0.854 [0.728–0.93]	0.604 [0.463–0.730]	0.708 [0.568–0.82]	0.792 [0.657–0.883]
LDA	AUC ROC	0.675 [0.583–0.767]	0.619 [0.523–0.716]	0.713 [0.626–0.8]	0.704 [0.616–0.79]	0.679 [0.588–0.77]	0.743 [0.66–0.826]	0.750 [0.668–0.832]
	Sensitivity	0.635 [0.529–0.73]	0.482 [0.379–0.587]	0.647 [0.541–0.740]	0.671 [0.565–0.76]	0.682 [0.577–0.772]	0.647 [0.541–0.74]	0.718 [0.614–0.802]
	Specificity	0.688 [0.547–0.801]	0.729 [0.590–0.834]	0.750 [0.612–0.851]	0.688 [0.547–0.8]	0.667 [0.525–0.783]	0.792 [0.657–0.88]	0.792 [0.657–0.883]

**Table 4:** Classification metrics for the *q*-range of 1.25–1.4 nm^−1^.

	Metrics	SAXS	SAXS Merid	SAXS Equat
LR	AUC ROC	0.679 [0.588–0.770]	0.677 [0.585–0.768]	0.630 [0.534–0.726]
	Sensitivity	0.624 [0.517–0.719]	0.671 [0.565–0.761]	0.565 [0.459–0.665]
	Specificity	0.771 [0.635–0.867]	0.708 [0.568–0.818]	0.688 [0.547–0.801]
LDA	AUC ROC	0.661 [0.568–0.754]	0.675 [0.583–0.766]	0.630 [0.534–0.726]
	Sensitivity	0.624 [0.517–0.719]	0.647 [0.541–0.740]	0.565 [0.459–0.665]
	Specificity	0.688 [0.547–0.801]	0.729 [0.590–0.834]	0.688 [0.547–0.801]

## Data Availability

The files with the XRD profiles are available at https://zenodo.org/records/22100448 (published 25 August 2026). The codes are available upon request.

## Supplementary Materials

The following supporting information can be downloaded at: https://www.mdpi.com/article/10.3390/cancers18203270/s1. Table S1: Demographic and clinical information. Table S2: Cancer stages. Table S3: Tumor sites. Table S4: Benign conditions. Table S5: Sample statistics. Figure S1: Participant flow diagram. Figure S2: Scattering profiles for all samples in the meridional direction of WAXS; Figure S3: Scattering profiles for all samples in the equatorial direction of WAXS; Figure S4: Scattering profiles for all samples in the meridional direction of SAXS; Figure S5: Scattering profiles for all samples in the equatorial direction of SAXS.
