# Supplementary material for: Feasibility of Cancer Detection Through X-Ray Diffraction of Human Nails
Source: Cancers (Basel). 2026 Oct 10;18(20):3270. doi: 10.3390/cancers18203270 (PMC13702462; doi:10.3390/cancers18203270)
Supplement: Supplementary file 1 [file cancers-18-03270-s001.zip › cancers-4562979-supplementary.pdf]

Characteristic	Controls	Cancer labelled
Patients / samples	48 / 92	85 / 138
Female, n (% of group)	33 (68.8%)	45 (52.9%)
Male, n (% of group)	14 (29.2%)	40 (47.1%)
Gender not recorded, n	1	0
Year of birth available, n	47	85
Year of birth, median (range)	1989 (1955–2021)	1960 (1942–1995)
Approximate age available, n	27	60
Approximate age, mean ± SD, years	36.3 ± 17.3	64.0 ± 9.5
Approximate age, median (IQR)	38 (27.5–46.5)	64.5 (60–70)
Approximate age range, years	4–70	30–83

Table S1: Demographic information.

Stage mentioned in the note	Patients	% of 85
I	30	35.3%
II	27	31.8%
III	23	27.1%
IV	5	5.9%

Table S2: Cancer stages.

Recorded tumour site	Patients	Patients %	Samples
Kidney	14	16.5%	26
Rectum	10	11.8%	16
Bladder	9	10.6%	18
Rectosigmoid	5	5.9%	8
Stomach	5	5.9%	6
Lung	5	5.9%	7
Bile duct	4	4.7%	6

Recorded tumour site	Patients	Patients %	Samples
Descending colon	4	4.7%	7
Breast	4	4.7%	7
Thyroid	3	3.5%	4
Endometrium	2	2.4%	2
Esophagus	2	2.4%	2
Ovary	2	2.4%	3
Liver	2	2.4%	3
Cecum	2	2.4%	2
Sigmoid colon	2	2.4%	3
Splenic flexure colon	2	2.4%	4
Testis	1	1.2%	2
Prostate	1	1.2%	2
Ureter	1	1.2%	2
Pancreas	1	1.2%	2
Skin	1	1.2%	1
Transverse colon	1	1.2%	2
Lower lip	1	1.2%	2
Ascending colon	1	1.2%	1
Total	85	100.0%	138

Table S3: Tumor sites.

Recorded benign condition	Patients	Samples
Renal or pararenal angiomyolipoma	2	4
Multinodular goiter	1	2
Pulmonary chondrohamartoma	1	2
Pulmonary fibrosis	1	2
Adrenal adenoma	2	4
Solid right testicular formation	1	2

Recorded benign condition	Patients	Samples
Total	8	16

Table S4: Benign conditions.

Samples per patient	Controls n (%)	Cancer labelled n (%)
1	9 (18.8%)	32 (37.6%)
2	34 (70.8%)	53 (62.4%)
3	5 (10.4%)	0 (0.0%)
Total patients	48	85

Recorded source code (finger)	Control samples	Cancer samples
R2	40	78
L2	47	60
R3	1	0
L4	1	0
RT1	1	0
Unknown	2	0
Total	92	138

Table S5: Sample statistics.

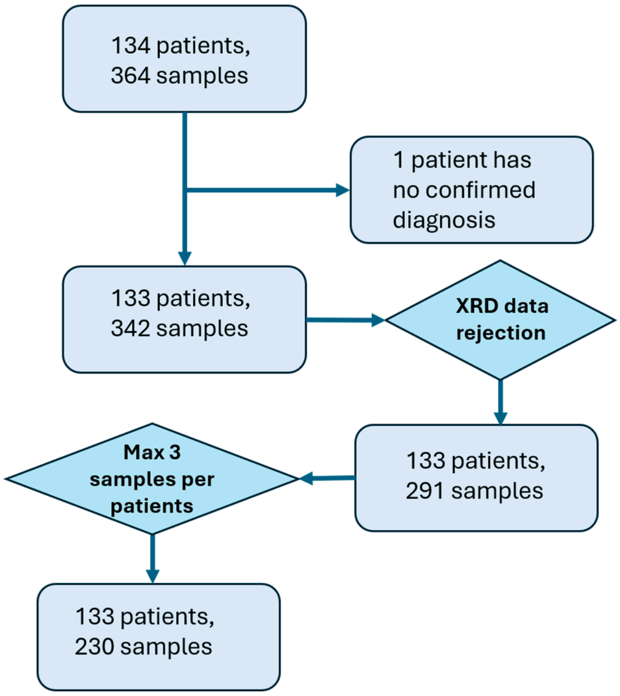

Figure S1: Participant flow diagram.

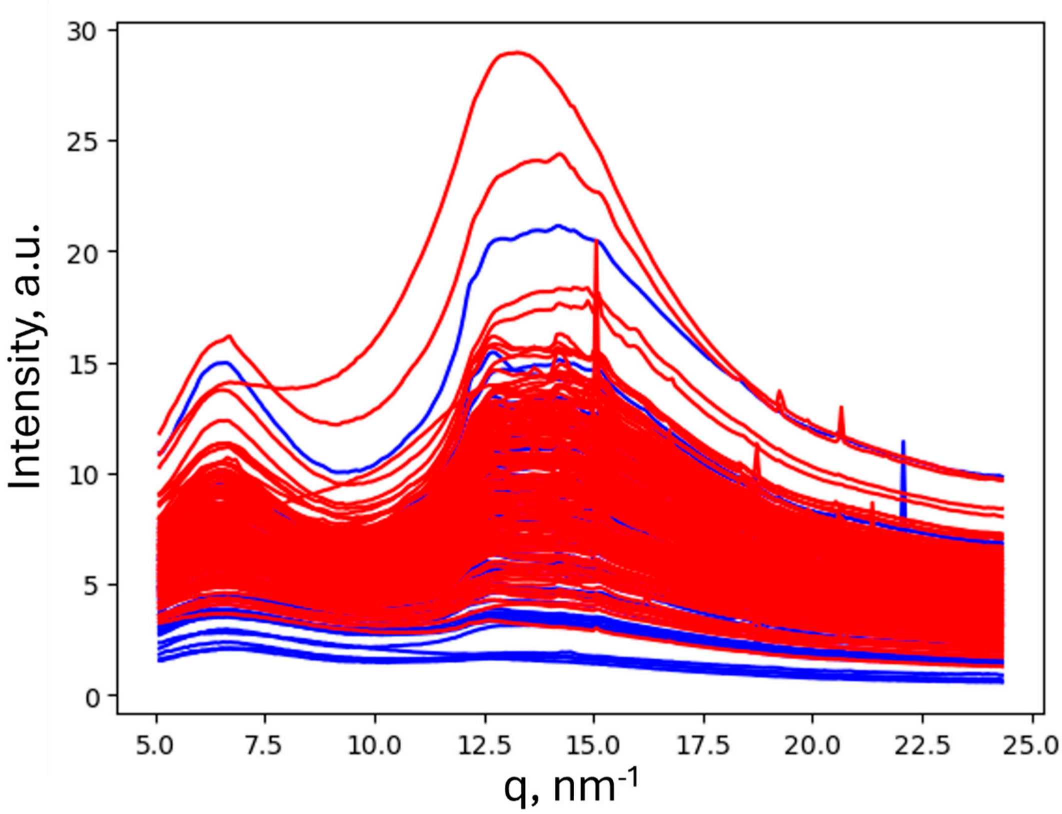

Figure S2: Scattering profiles for all samples in the meridional direction of WAXS. Blue: control. Red: cancer.

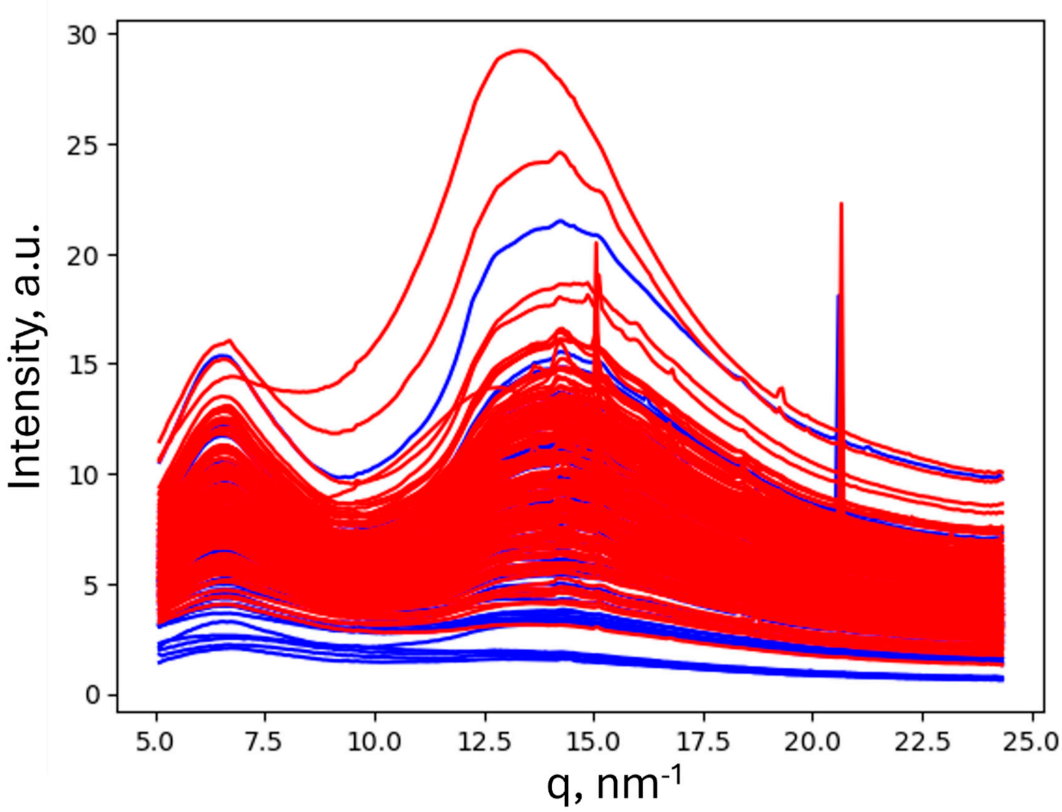

Figure S3: Scattering profiles for all samples in the equatorial direction of WAXS. Blue: control. Red: cancer.

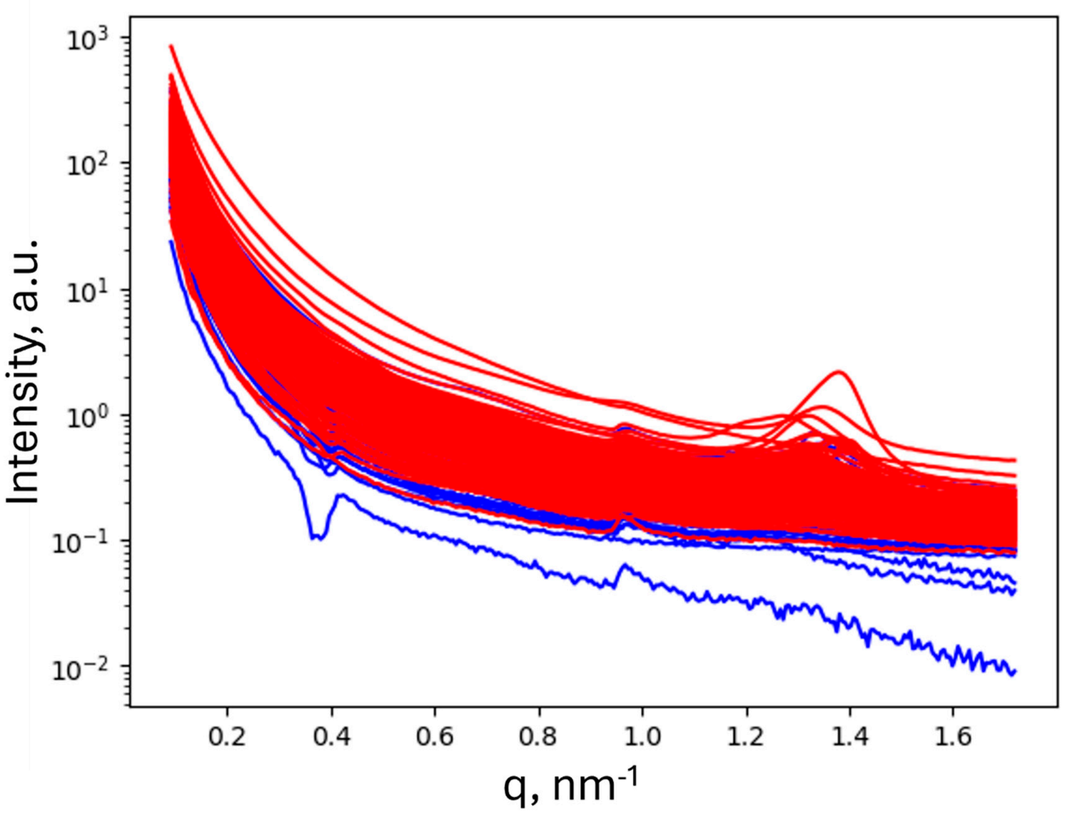

Figure S4: Scattering profiles for all samples in the meridional direction of SAXS. Blue: control. Red: cancer.

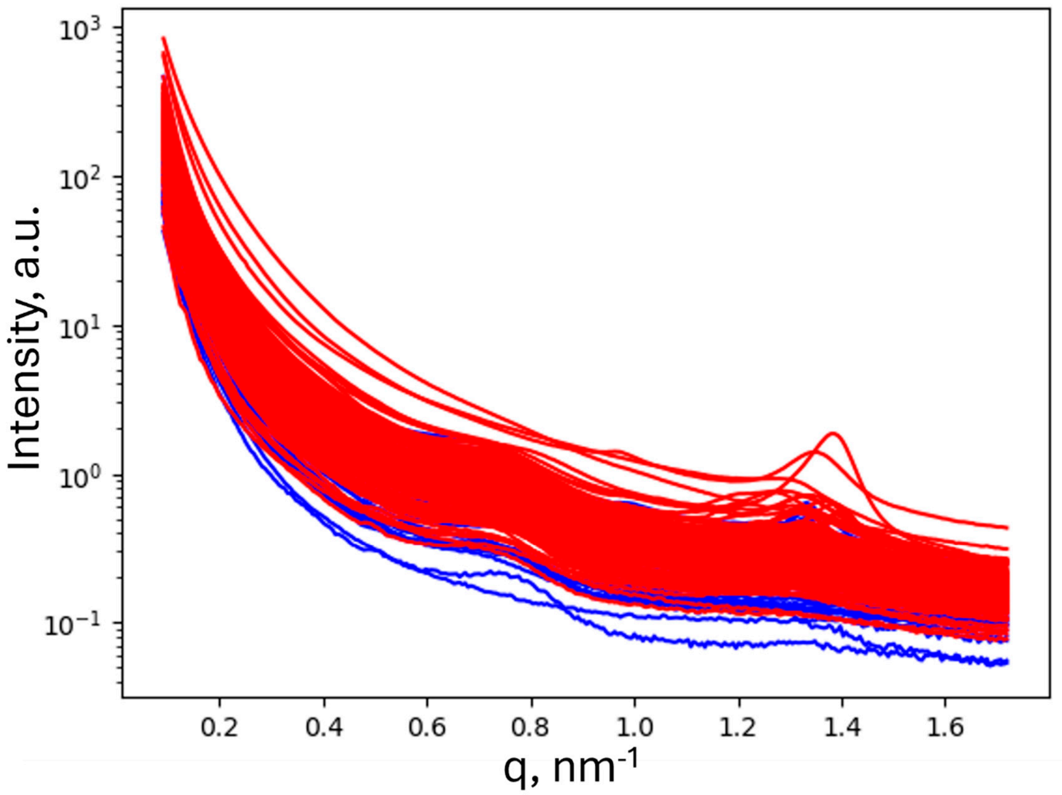

Figure S5: Scattering profiles for all samples in the equatorial direction of SAXS. Blue: control. Red: cancer.
